# Supplementary material for: The causal role of circulating inflammatory markers in osteoporosis: a bidirectional Mendelian randomized study
Source: Front Immunol. 2024 Jul 18;15:1412298. doi: 10.3389/fimmu.2024.1412298 (PMC11291241; doi:10.3389/fimmu.2024.1412298)
Supplement: Supplementary file 1 [file DataSheet_1.zip › supplementary material/OSTEOPOROSIS-CIM/GCST90274842.tsv.gz_leave_one_out.pdf]

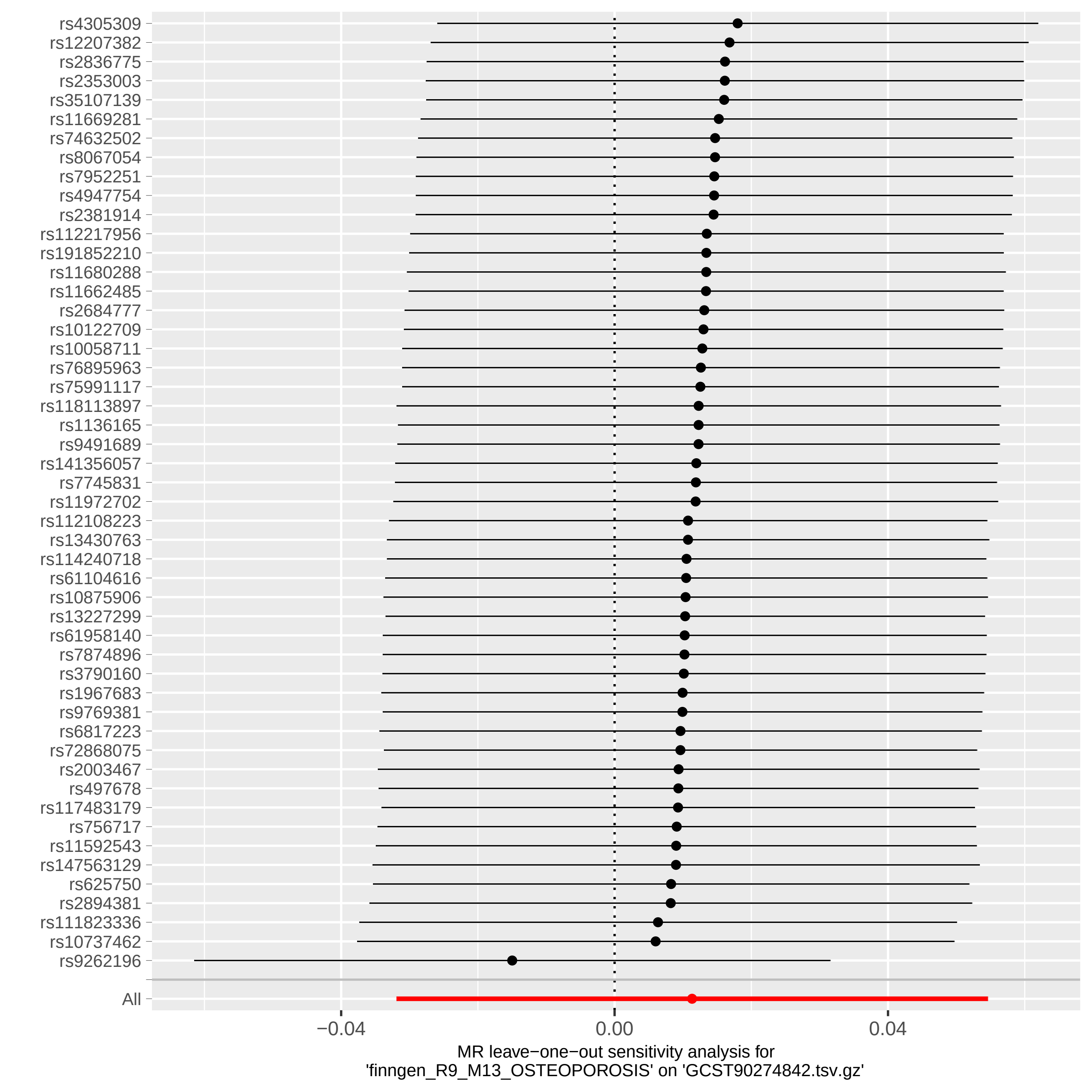
rs4305309
rs12207382
rs2836775
rs2353003
rs35107139
rs11669281
rs74632502
rs8067054
rs7952251
rs4947754
rs2381914
rs112217956
rs191852210
rs11680288
rs11662485
rs2684777
rs10122709
rs10058711
rs76895963
rs75991117
rs118113897
rs1136165
rs9491689
rs141356057
rs7745831
rs11972702
rs112108223
rs13430763
rs114240718
rs61104616
rs10875906
rs13227299
rs61958140
rs7874896
rs3790160
rs1967683
rs9769381
rs6817223
rs72868075
rs2003467
rs497678
rs117483179
rs756717
rs11592543
rs147563129
rs625750
rs2894381
rs111823336
rs10737462
rs9262196
All
−0.04
0.00
0.04
MR leave−one−out sensitivity analysis for
'finngen_R9_M13_OSTEOPOROSIS' on 'GCST90274842.tsv.gz'
